# Real-world data to build explainable trustworthy artificial intelligence models for prediction of immunotherapy efficacy in NSCLC patients

**DOI:** 10.3389/fonc.2022.1078822

**Published:** 2023-01-23

**Authors:** Arsela Prelaj, Edoardo Gregorio Galli, Vanja Miskovic, Mattia Pesenti, Giuseppe Viscardi, Benedetta Pedica, Laura Mazzeo, Achille Bottiglieri, Leonardo Provenzano, Andrea Spagnoletti, Roberto Marinacci, Alessandro De Toma, Claudia Proto, Roberto Ferrara, Marta Brambilla, Mario Occhipinti, Sara Manglaviti, Giulia Galli, Diego Signorelli, Claudia Giani, Teresa Beninato, Chiara Carlotta Pircher, Alessandro Rametta, Sokol Kosta, Michele Zanitti, Maria Rosa Di Mauro, Arturo Rinaldi, Settimio Di Gregorio, Martinetti Antonia, Marina Chiara Garassino, Filippo G. M. de Braud, Marcello Restelli, Giuseppe Lo Russo, Monica Ganzinelli, Francesco Trovò, Alessandra Laura Giulia Pedrocchi

**Affiliations:** ^1^ Medical Oncology Department, Fondazione IRCCS Istituto Nazionale Tumori, Milan, Italy; ^2^ Department of Electronics, Information and Bioengineering, Politecnico di Milano, Milan, Italy; ^3^ Niguarda Cancer Center, Grande Ospedale Metropolitano Niguarda, Milan, Italy; ^4^ Oncology Department, University of Milan, Milan, Italy; ^5^ Medical Oncology Unit, Department of Precision Medicine, University of Campania “Luigi Vanvitelli”, Naples, Italy; ^6^ Medical Oncology Unit, Policlinico San Matteo Fondazione IRCCS, Pavia, Italy; ^7^ Department of Electronic System, Aalborg University, Copenhagen, Aalborg, Denmark; ^8^ Thoracic Oncology Program, Section of Hematology/Oncology, University of Chicago, Chicago, IL, United States

**Keywords:** non-small cell lung cancer, immunotherapy, machine learning, explainable artificial intelligence, treatment

## Abstract

**Introduction:**

Artificial Intelligence (AI) methods are being increasingly investigated as a means to generate predictive models applicable in the clinical practice. In this study, we developed a model to predict the efficacy of immunotherapy (IO) in patients with advanced non-small cell lung cancer (NSCLC) using eXplainable AI (XAI) Machine Learning (ML) methods.

**Methods:**

We prospectively collected real-world data from patients with an advanced NSCLC condition receiving immune-checkpoint inhibitors (ICIs) either as a single agent or in combination with chemotherapy. With regards to six different outcomes - Disease Control Rate (DCR), Objective Response Rate (ORR), 6 and 24-month Overall Survival (OS6 and OS24), 3-months Progression-Free Survival (PFS3) and Time to Treatment Failure (TTF3) - we evaluated five different classification ML models: CatBoost (CB), Logistic Regression (LR), Neural Network (NN), Random Forest (RF) and Support Vector Machine (SVM). We used the Shapley Additive Explanation (SHAP) values to explain model predictions.

**Results:**

Of 480 patients included in the study 407 received immunotherapy and 73 chemo- and immunotherapy. From all the ML models, CB performed the best for OS6 and TTF3, (accuracy 0.83 and 0.81, respectively). CB and LR reached accuracy of 0.75 and 0.73 for the outcome DCR. SHAP for CB demonstrated that the feature that strongly influences models’ prediction for all three outcomes was Neutrophil to Lymphocyte Ratio (NLR). Performance Status (ECOG-PS) was an important feature for the outcomes OS6 and TTF3, while PD-L1, Line of IO and chemo-immunotherapy appeared to be more important in predicting DCR.

**Conclusions:**

In this study we developed a ML algorithm based on real-world data, explained by SHAP techniques, and able to accurately predict the efficacy of immunotherapy in sets of NSCLC patients.

## Introduction

Over the past decade, immunotherapy (IO) has significantly changed the therapeutic landscape of lung cancer, particularly non-small cell lung cancer (NSCLC) (1, 2). The median overall survival (mOS) of patients with advanced non-oncogene addicted NSCLC improved from approximately 12 months in the chemotherapy era to about 24 months with the advent of IO (3). The 5-year survival rate increased from 16% with chemotherapy alone to 32% with the addition of IO (4). Despite these important results, only 30-50% of patients achieve long-term benefits from IO (5–7).

In clinical practice, Programmed Death-Ligand 1 (PD-L1) is as of now the only biomarker used to predict response to ICIs as a higher PD-L1 expression is generally associated with the possibility of response to IO. However, the observation that approximately 40% of patients with a high expression of PD-L1 do not benefit from therapy leads to the conclusion that its predictive ability is not satisfactory (8). Several biomarkers that could provide an alternative are currently being studied, some of them focused on tumor characteristics - including tumor mutational burden (TMB), tumor microenvironment (TME), microsatellite instability (MSI), somatic mutations - and others on the patient’s characteristics, including performance status (PS), BMI, smoking history, blood count/blood tests, microbiome, corticosteroid use, more still regard radiomics or their combination in different scores (9, 10). Indeed, the complexity of the immune response is difficult to capture with a single biomarker, therefore the most effective option would be to consider a combination of all biomarkers simultaneously to obtain the whole picture.

In oncology, new technologies such as Artificial Intelligence (AI) and Machine Learning (ML) methodologies are gaining increasing attention, as they are able to analyze complex nonlinear behaviors, from multidimensional data, essential for clinical practice given the need for integrated real-world and multi-omics data analysis. ML merges patient and tumor data and thereby increases the accuracy of prediction biomarkers (11) leading to the personalization of treatment and the selection of patients who can benefit from IO. Johannet et al. (12) used Deep Leaning (DL) to stratify patients receiving IO for advanced melanoma into those with high and low risk of disease progression, selecting features according to both histological characteristics and clinic-demographic data, generating a model with an AUC of 0.80.

Eventually, ML could help increase the chance of survival and reduce immune-related toxicities and healthcare costs. However, ML methods only reveal the input data and the produced output, but it is currently not possible to assess how the algorithms have generated a specific result, the so-called “black-box” issue. Since in the medical field, particularly in oncology, it is crucial to understand how the result was achieved, trustworthy Explainable AI (XAI) has to be the way forward (13).

This study aims to integrate clinical, radiological and haematochemical features at the baseline of IO treatment, to develop an explainable white box model able to predict the response and efficacy of IO in patients with advanced NSCLC – in turn this will improve the personalization of the treatment and provide support to the clinical decision-making process (14).

## Materials and methods

### Study population

The study presented here was a prospective observational study (APOLLO Study, INT 22_15) in advanced NSCLC patients treated between January 2015 and Jun 2021 in a single Italian institution, Fondazione IRCCS Istituto Nazionale Tumori (Milan).

**Figure 1:**
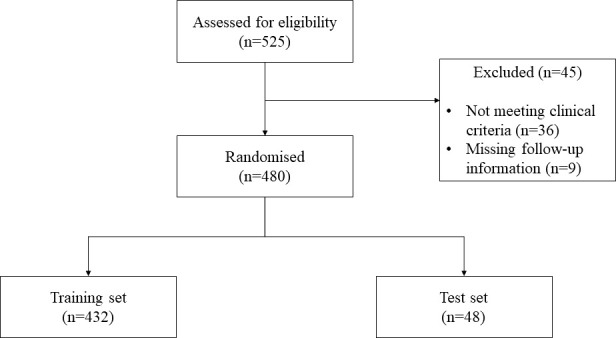
Flowchart of participants progress through the study.

Eligibility criteria were: (1) patients with cytologically or histologically confirmed diagnosis of stage IV or recurrent NSCLC; (2) age ≥ 18 years; (3) receiving at least one administration of first or further-line ICIs either alone or in combination with chemotherapy; (4) available data about efficacy outcomes with study treatment: Objective Response Rate (ORR), Disease Control Rate (DCR) as best response; Overall Survival (OS), Progression Free Survival (PFS) and Time to Treatment Failure (TTF). The CONSORT flow diagram is shown in **Figure 1**.

For the study, demographic, medical history and molecular data, treatment response, and survival follow-up were collected to create a model for predicting response to IO in patients with advanced NSCLC.

The study (Apollo, INT 22_15) was approved by the Ethical Committee of “Fondazione IRCCS Istituto Nazionale Tumori”, and all patients have signed the informed consent. It was conducted according to Good Clinical Practice guidelines and the Declaration of Helsinki principles.

### Radiological response evaluation

Baseline radiological evaluation included a total-body CT scan performed within 30 days before the start of IO. The subsequent ones were performed every 9-12 weeks according to clinical practice or earlier in case of clinical suspicion of progression or according to medical judgment.

Six categories of radiological response were considered in assessing tumor response to treatment, of whom four were included in the Response Evaluation Criteria for Solid Tumors version 1.1 (RECIST1.1): Complete Response (CR), Partial Response (PR), Stable Disease (SD), and Progression Disease (PD). Hyper-Progressive Disease (HPD) category was defined according to the criteria of Lo Russo et al., whereas patients who died or lost to follow-up before the first radiological assessment of response were included in Not Evaluable (NE) category (15, 16).

### Treatment administration

IO treatment was administered intravenously (IV) as monotherapy or in combination with chemotherapy. IO regimens included: anti PD-1 as Nivolumab, at a dose of 3 mg/kg or a flat dose of 240 mg every 2 weeks (w), and pembrolizumab at a flat dose of 200 mg in the first Line or a dose of 2 mg/kg every 3w in further lines or 400 mg dose flat every 6w; anti PD-L1 as durvalumab at a dose of 10 mg/kg every 2w, atezolizumab 1200 mg every 3w, and avelumab 10mg/kg every 2w; anti-CTLA-4 tremelimumab 10mg/kg every 2w; anti-TGFbeta M7824 1200mg every 2w.

Combination treatments included platinum-based therapy (carboplatin AUC5 and Cisplatin at a dose of 75 mg/mq) in combination with either pemetrexed and paclitaxel at a dose 500 mg/mq and 200 mg/mq, respectively and pembrolizumab 200 mg every 3w for 4 cycles. Maintenance therapy followed with pemetrexed plus pembrolizumab or pembrolizumab alone every 3w based on non-squamous or squamous histology, respectively.

Immunotherapy treatment was administered until the occurrence of intolerable toxicity, PD or death. In some cases, IO was administered beyond radiological progression, according to physician evaluation.

### Statistical analysis

The descriptive statistical analysis of the data, such as demographic, clinical, biochemical, and radiological variables, was performed using the software SPSS v. 28.0. Group comparisons were performed using two-sided Mann–Whitney U-tests in Python script.

### Machine learning workflow

**Figure 2:**
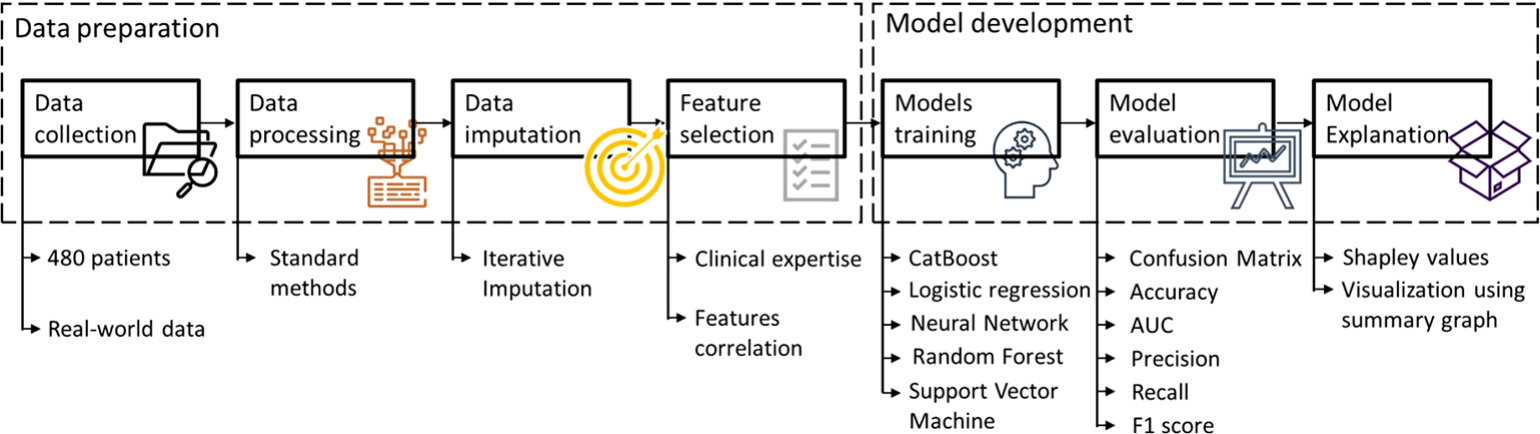
Methodology workflow for developing different ML/XAI models. *AUC, area under the curve*.

The methodology workflow for developing different ML/XAI models is reported in **Figure 2**.

### Data processing and imputation

After data collection, the dataset was divided into a training and a test set, in a ratio of 9:1, respectively. Accordingly, the training set contained 432, and the test set 48 patients. Imputation of the missing data was performed using the Iterative Imputation algorithm (17). This Multivariate approach assigns imputed values by solving a linear regression problem performed on other features. Each feature containing missing values is, in turn, considered the target of the regression model. The imputation algorithm is fitted on the training set and then applied to both training and test dataset. After imputation, all the continuous features have been rescaled to values between 0 and 1.

### Feature selection

The feature selection was performed using two different approaches: one based on clinical expertise, and another based on the correlation between features. First, we manually removed (a) features that could not be collected at the baseline of IO treatment, (b) features that represented the same concept but were expressed differently, and (c) features that had more than 25% of missing data. After this the number of features was reduced to 28 based on literature and clinical experience. To avoid high correlated features in input, in the case a pair of features showed a linear correlation with absolute value larger than 0.8, we removed one of them. The final list of 27 features is given in **Table 1**.

**Table 1:** Selected features.

Feature type	Feature	Feature description
**Clinical feature**	Age	Age at Baseline of IO
	Sex	Sex: female (0) or male (1)
	Smoke	Smoking status: non-smoker (0) or smoker (1)
	ECOG PS	ECOG Performance Status, from 0 to 5, where 0 is the best and 5 the worst status (dead)
	BMI	BMI at the Baseline of IO
**Radiological**	Liver mets	Liver Metastasis at Baseline of IO
	Brain mets	Brain Metastasis at Baseline of IO
	Bone mets	Bone Metastasis at Baseline of IO
	Lymph nodes mets	Lymph nodes Metastasis at Baseline of IO
	Adrenal mets	Adrenal Metastasis at Baseline of IO
	Pleura mets	Pleura Metastasis at Baseline of IO
**Laboratory exams**	ALC	Absolut Leukocytes count at baseline of IO
	ANC	Absolute Neutrophils count at baseline of IO
	AMC	Absolute Monocytes count at baseline of IO
	ALyC	Absolute Lymphocytes count at baseline of IO
	NLR	Neutrophils to Lymphocytes ratio at baseline of IO
	LDH	Lactate Dehydrogenase at baseline of IO
**Staging**	TNMd	TNM staging at diagnosis
	TNMio	TNM staging at baseline of IO
	T	Tumor Stage at Baseline of IO
	N	Node Stage at baseline of IO
**Treatment information**	IO/IOCT	Indicates if a patient received just Immunotherapy (0) or Immunotherapy with Chemotherapy (1)
	Nr Line IO	Number line of IO
	Surgery	Surgery (0 = No, 1= Yes)
	RT	Radiotherapy prior IO
**Tumor characteristic**	Histology	Indicates if the tumor type is Squamous (1) or not (0)
	PDL1	Value of PD-L1 divided in 3 classes:>1 (1), 1>PD-L1<49 (2) and ≥50 (3)

### Outcomes

**Figure 3:**
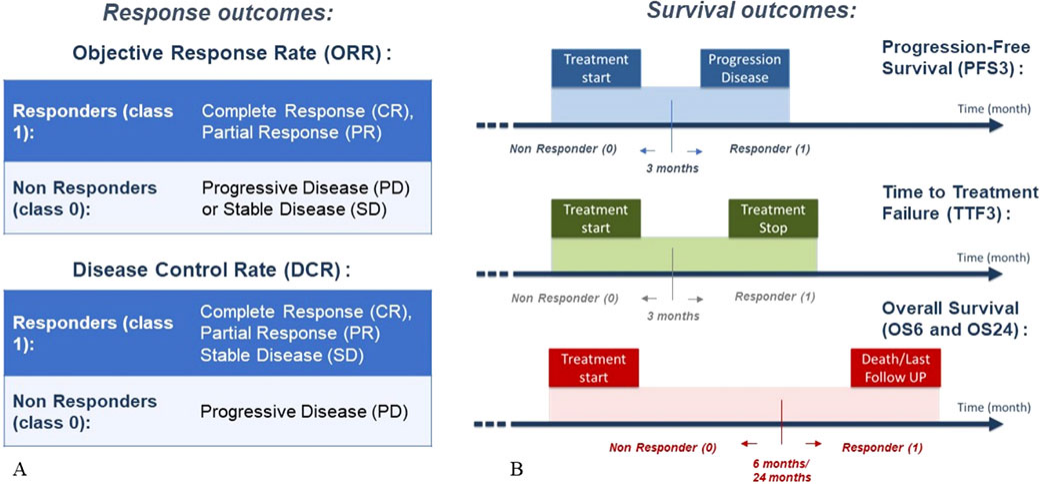
Endpoints of the study with descriptions, in terms of response outcomes **(A)** and survival outcomes **(B)**.

We used six different outcomes: DCR, ORR, 6-months OS (OS6), 24-months OS (OS24), 3-months PFS (PFS3) and 3-months TTF (TTF3). The list of outcomes, with the description, is shown in **Figure 3**. OS6 and OS24 were both used to develop an ML algorithm to identify patients who experienced a fast death (OS6 = 0) compared to a second OS cut-off (OS24 = 1) corresponding to long-survival patients. For PFS and TTF, the same cut-off of 3 months was selected to build an algorithm able to identify patients who will progress immediately after IO (TTF< 3 months). Outcomes ORR and OS24 were highly imbalanced, meaning that one class has very low proportions in the dataset compared to the other class.

### Machine learning models

Since we previously chose a threshold value for the survival outcomes, predicting all the outcomes listed in **Figure 3** is a binary classification problem. In this study, we used five different ML classification techniques: Logistic Regression (LR), feedforward Neural Network (NN), Support Vector Machine (SVM), Random Forest (RF), and CatBoost (CB). The development of the first four ML models is described in detail in our latest publication (11). In this work we wanted to compare the performance of CB model (18) with the respect to these state-of-the-art models. For the CB model, we used the Python implementation (v 0.26), provided by Dorogush et al. (18) and Prokhorenkova et al. (19) CB model parameters were optimized using Grid Search. These were tested using a 10-fold cross-validation targeted to maximize the validation F1 score. In the case of outcomes with imbalanced classes (OS24 and ORR), we computed the class weight that was further included in the model. The main reasons for selecting the CB model among other similar techniques are examples of CB’s successful application in oncological studies (20–22) where it outperformed the other gradient models. The efficacy of models was evaluated and compared using the following performance metrics: confusion matrix, accuracy (ACC), Precision, Recall, F1-score and Area Under the Curve (AUC).

### Explainable AI methods

To understand how models yielded their prediction, we used SHapley Additive exPlanations (SHAP) values as proposed by Lundberg and Lee (23). SHAP is among the most frequently used algorithms applied in XAI. It allows assigning a value, the so-called “Shapley value”, to each feature based on how much it affects the output prediction. The Shapley value expresses the contribution of each feature to a given prediction compared to the average prediction (24). SHAP values were computed for the test set for all models using the method provided by Lundberg and Lee (23).

## Results

### Patient characteristics

A total of 480 patients with advanced NSCLC (96 squamous and 384 non-squamous, 20% and 80% of the total, respectively) were included in the study. The median age was 67 years (range: 27-89 years) with 202 (42%) patients older than 70. The majority of patients were male (n=298, 62%), smokers or former smokers (n=406, 84.6%) and received IO alone (407, 85%). 13.9% of patients presented an Eastern Cooperative Oncology Group (ECOG) performance status (PS) ≥ 2 (only two patients had PS 3 at baseline). Immunotherapy was administered as the First Line of treatment in 247 patients (51.5%), while 233 (48.5%) received IO in further lines: 146 as the second Line (30.4%), 57 in the third Line (11.9%) and 30 (6.2%) in subsequent lines. Patients’ characteristics are shown in **Table 2**.

**Table 2:** Patient characteristics used in this study.

Characteristics	Entire cohort (n=480)	Training set (n=432)	Test set (n=48)
	n (%)	n (%)	n (%)
**Age, median (range)**	67 (27 – 89)	67 (27 – 89)	65 (31 - 84)
Sex			
Female	182 (37,91%)	162 (37,5%)	20 (41,66%)
Male	298 (62,09%)	270 (62,5%)	28 (58,33%)
Treatment			
IO	407 (84,79%)	366 (84,72%)	41 (85,41%)
IO/CT	73 (15,21)	66 (15,28%)	7 (14.58%)
Histology			
Non-Squamous	384 (80%)	342 (79.16%)	42 (87,5%)
Squamous	96 (20%)	90 (20.84%)	6 (12,5%)
ECOG performance status			
ECOG 0	160 (33,33%)	146 (33,79%)	14 (29,16%)
ECOG 1	253 (52,71%)	229 (53,01%)	24 (50%)
ECOG ≥2	67 (13,95%)	57 (13,19%)	10 (20,83%)
PD-L1 expression			
<1	119 (24,79%)	109 (23,14%)	10 (20,83%)
1-49	135 (28,12%)	124 (28,70%)	11 (22,92%)
≥50	110 (22,91%)	97 (22,47%)	13 (27,08%)
DCR			
Class 0 (PD)	233 (48,54%)	210 (48,66%)	23 (47,91%)
Class 1 (CR/PR/SD)	247 (51,45%)	222 (51,34%)	25 (52,08%)
OS6			
Class 0 (<6month)	195 (40,62%)	173 (40,04%)	22 (45,83%)
Class 1 (≥6months)	285 (59,37%)	259 (59,96%)	26 (54,17%)
PFS			
Class 0 (<3month)	234 (48,75%)	210 (43,75%)	24 (50%)
Class 1 (≥3months)	246 (51,25%)	222 (56,25%)	24 (50%)

### Machine learning analysis

In **Tables 3**–**5**, we reported all the results obtained with different ML models for the most significant outcomes: DCR, OS6, and TTF3, respectively. In contrast, results for the other three outcomes (ORR, OS24, PFS) were included in **Tables S2–S4** in the Supplementary information section, as are all the features that were selected for models LR, NN, RF and SVM, listed in **Table S1**.

**Table 3:** Performance of classification models on the test dataset; outcome – DCR.

Outcome	Model	Features	Class	N. class	Precision	Recall	F1	ACC	AUC
**DCR** **Class 0** **(PD)** **233 patients** **Class 1** **(SD+PR+CR)** **247** **patients**	**CB**	**27**	**0**	**23**	**0.70**	**0.83**	**0.76**	**0.75**	**0.75**
			**1**	**25**	**0.81**	**0.68**	**0.74**		
	**LR**	**10**	**0**	**23**	**0.69**	**0.78**	**0.73**	**0.73**	**0.77**
			1	25	0.77	0.68	0.72		
	NN	11	0	23	0.58	0.61	0.60	0.60	0.63
			1	25	0.62	0.60	0.61		
	RF	7	0	23	0.64	0.78	0.70	0.68	0.71
			1	25	0.75	0.60	0.67		
	SVM	15	0	23	0.52	0.65	0.57	0.54	0.58
			1	25	0.58	0.44	0.50		

ACC, testing accuracy; AUC, area under the curve; DCR, disease control rate; PD, progressive disease; SD, stable disease; PR, partial response; CR, complete response; N. class, number of patients in both classes for a test set. Bold is the best performing model for this outcome.

**Table 4:** Performance of classification models on the test dataset; outcome – OS6.

Outcome	Model	Features	Class	N. class	Precision	Recall	F1	Acc.	AUC
**OS6** **Class 0** **(OS<6m)** **195** **patients** **Class 1** **(OS≥6m)** **285** **patients**	**CB**	**27**	**0**	**22**	**0.85**	**0.77**	**0.81**	**0.83**	**0.82**
			**1**	**26**	**0.82**	**0.88**	**0.85**		
	LR	8	0	22	0.71	0.54	0.62	0.69	0.79
			1	26	0.68	0.81	0.73		
	NN	10	0	22	0.64	0.41	0.50	0.63	0.79
			1	26	0.62	0.81	0.70		
	RF	4	0	22	0.68	0.59	0.63	0.69	0.76
			1	26	0.69	0.77	0.73		
	SVM	5	0	22	0.48	0.54	0.51	0.52	0.47
			1	26	0.57	0.50	0.53		

ACC, testing accuracy; AUC, area under the curve; OS, 6-months overall survival; N. class, number of patients in both classes for a test set. Bold is the best performing model for this outcome.

**Table 5:** Performance of classification models on the test dataset; outcome – TTF3.

Outcome	Model	Features	Class	N. class	Precision	Recall	F1	Acc.	AUC
**TTF3** **Class 0** **(TTF<3m)** **213** **patients** **Class 1** **(TTF3≥6m)** **267** **patients**	**CB**	**27**	**0**	**23**	**0.79**	**0.83**	**0.81**	**0.81**	**0.81**
			**1**	**25**	**0.83**	**0.80**	**0.82**		
	LR	9	0	23	0.76	0.70	0.73	0.75	0.79
			1	25	0.74	0.80	0.78		
	NN	10	0	23	0.74	0.74	0.74	0.75	0.78
			1	25	0.76	0.76	0.76		
	RF	5	0	23	0.57	0.57	0.57	0.58	0.65
			1	25	0.60	0.60	0.60		
	SVM	11	0	23	0.53	0.78	0.63	0.56	0.51
			1	25	0.64	0.36	0.46		

ACC, testing accuracy; AUC, area under the curve; TTF3, 3-months’ time to treatment failure; N. class, number of patients in both classes for a test set. Bold is the best performing model for this outcome.

As reported in **Table 3**, the best results for the DCR outcome were achieved using CB and LR models. Accuracy and AUC for the CB model were 0.75, while F1 scores were 0.76 and 0.74 for classes 0 and 1, respectively. Similarly, the LR model achieved an accuracy of 0.73 and a slightly higher AUC (0.77) compared to the CB model, while F1 scores were 0.73 and 0.72 for classes 0 and 1, respectively. For the OS6 outcome (**Table 4**), CB achieved the best results concerning all evaluation metrics, reaching an accuracy of 0.83, AUC of 0.81 and F1 score of 0.81 and 0.85 for classes 0 and 1, respectively. **Table 5** summarizes the results obtained for the outcome TTF3. CB again achieved the best results in terms of accuracy (0.81), AUC (0.81) and F1 score for class 0 (0.81) and class 1 (0.82).

**Figure 4:**
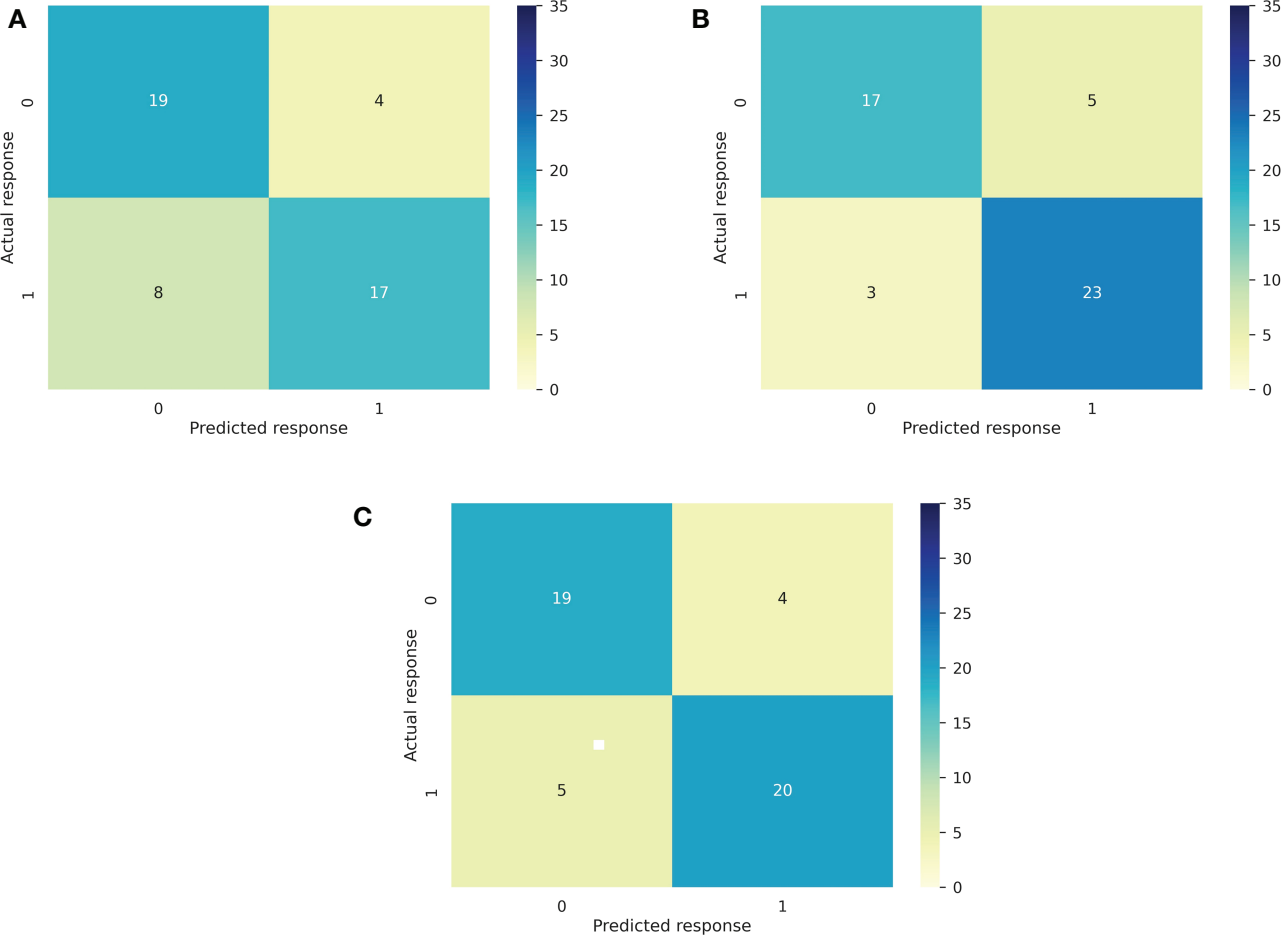
Confusion Matrixes for the CB model for main outcomes: DCR **(A)**, OS6 **(B)**, and TTF3 **(C)**.

In **Figure 4** we report the Confusion Matrixes for the CB model for outcomes DCR, OS6, and TTF3, respectively. Confusion Matrixes for other outcomes (ORR, OS24 and PFS3) are reported in **Figures 1S A–C in Supplementary information**.

### Explainable AI

We compared SHAP values for different models and outcomes using a summary plot: features are shown along the y axis in descending order based on their importance, which is given by the average of their absolute SHAP value. Each colored dot corresponds to a patient; the color ranges from blue to red, depending on the absolute value of that feature for the patient. A SHAP value of 0 represents the absence of influence on the outcome. In contrast, a positive or negative SHAP value defines a positive or negative association with the explored outcome (24, 25).

**Figure 5:**
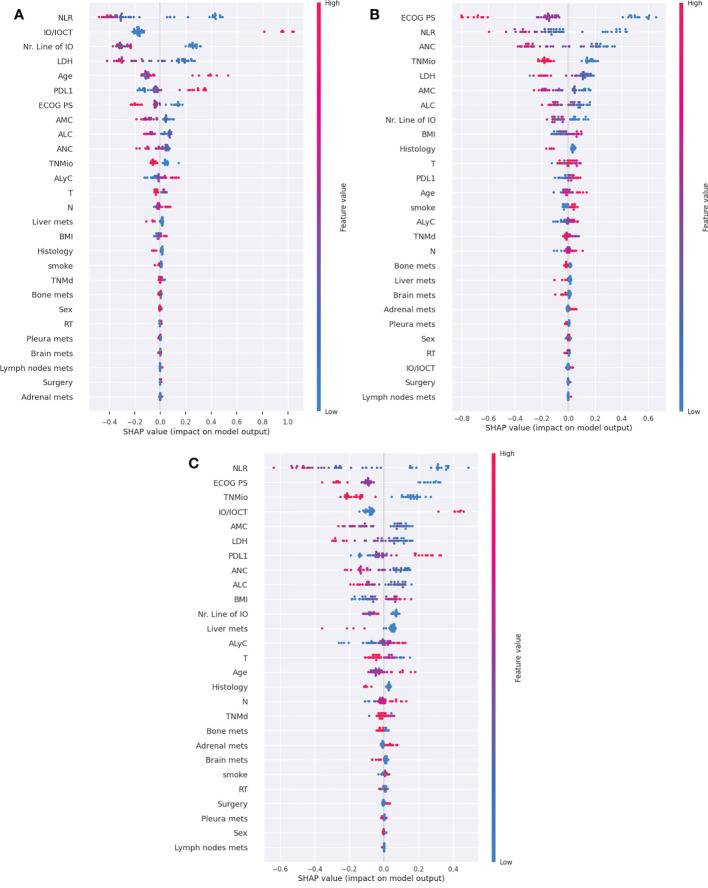
SHAP summary plots for the CB model for three main outcomes: DCR **(A)**, OS6 **(B)** and TTF3 **(C)**. IO/IOCT, immuno-oncologic treatment/immuno-oncologic and chemotherapy treatment; PDL-1, programmed death ligand-1; ECOG PS, eastern cooperative oncology group performance status; NLR, Neutrophil-to-lymphocyte ratio; AMC, absolute monocytes count; ALC, absolute leucocytes count; ANC, absolute neutrophils count; ALyC, absolute lymphocytes count; TNMd, TNM staging at diagnosis; TNMio, TNM staging at baseline of IO; BMI, body mass index; RT, radiotherapy.

In **Figure 5** we have shown SHAP summary plots for the CB model for three outcomes, TTF3, DCR and OS6, while summary plots for the remaining outcomes (ORR, OS24 and PFS3) are presented in the Supplementary information (**Figure S2**), together with summary plots generated for other models (**Figures S3–6**).

Among the five most important features for predicting the non-responders for the DCR outcome, as reported in **Figure 5A**, were the following: high values of neutrophil-to-lymphocyte ratio (NLR), single agent IO (compared to combination with IO and chemotherapy), a higher Line of IO therapy (i.e., if it was given as a further line of therapy), a high value of lactate dehydrogenase (LDH) level, and younger age; while low values of PD-L1 are ranked sixth by importance. As shown in the summary plot for the OS6 outcome (**Figure 5B**), the five most important features are ECOG PS, NLR, LDH, the absolute value of neutrophiles and TNM staging at the IO baseline. High values of the features above correlate negatively with response to IO, leading to class 0 (OS<6 months). For the outcome TTF3, the SHAP summary plot (**Figure 5C**) showed that the most important features are: NLR, ECOG PS, TNM staging at the baseline, IO/IOCT and Monocytes. High values for NLR, Line of IO, TNMio staging, and monocytes yield predictions towards TTF3<3months (class 0). Once again, similarly to what has been presented for the DCR outcome, patients could benefit from IO in combination with chemotherapy, as the feature is pushing the prediction towards TTF3≥3 months (class 1). For the TTF3 outcome, PD-L1 is in seventh place by importance.

### Comparing features selected from different ML models

**Table 6** lists the six most important features, selected by Shapley, for the CB and LR models for the DCR, OS6 and TTF3 outcomes. As shown in **Table 6**, NLR and ECOG PS were the most represented and important features across the models. Treatment type (IO/IOCT) was found to have more influence on the prediction of DCR and TTF3 compared to OS6. The combination of IO and chemotherapy benefits patients in both cases and favors the prediction of class 1 (responders) and TTF ≥ 3 months.

**Table 6:** List of the most important features for DCR, OS6 and TTF3.

Outcomes	Model	1	2	3	4	5	6
**DCR** **Class 0:** **PD** **Class 1: SD+PR+CR**	**CatBoost**	**NLR**	**IO/IOCT**	**Line of IO**	**LDH**	**Age**	**PD-L1**
	**Logistic** **Regression**	**IO/IOCT**	**PD-L1**	**NLR**	**TNMio**	**ECOG PS**	**RT**
**OS6** **Class 0/1: <6/≥6 months**	**CatBoost**	**ECOG PS**	**NLR**	**ANC**	**TNMio**	**LDH**	**AMC**
	**Logistic** **Regression**	**ECOG PS**	**Line of IO**	**TNMio**	**NLR**	**Histology**	**AMC**
**TTF3** **Class 0/1: <3/≥3 months**	**CatBoost**	**NLR**	**ECOG PS**	**TNMio**	**IO/IOCT**	**AMC**	**LDH**
	**Logistic** **Regression**	**NLR**	**IO/IOCT**	**ECOG PS**	**PD-L1**	**TNMio**	**AMC**

IO/IOCT, Immunotherapy alone vs chemo-immunotherapy combination.

The position of the features from 1 to 6 is determined based on the most important feature in terms of Shap Value. The red color is assigned to those features where a high value led to a negative correlation with DCR, OS6 or TTF3 (Red high value (Class 0); Green color is assigned to those features in which a high value positively correlates with DCR and OS6 (Green high value (Class 1). For IO/IOCT, the high value means the chemo and IO combination therapy.

We compared the distribution of the most important features (**Table 6** for the CatBoost model) between Responders (DCR=1) and Non-Responders (DCR=0) in the test set for each outcome. For the DCR, only the NLR feature has a statistically significant difference (P=0.004) in the distribution between responders and non-responders. While for OS6 and TTF3 only PS at the baseline has statistically significant distribution differences between the groups, P=0.0003 and P=0.004, respectively. The CatBoost model is capable of capturing nonlinear interaction effects between the features, which can presumably explain why most of the features that identify as important for CatBoost prediction using SHAP do not have a statistically significant difference between the two groups, responders vs non-responders.

## Discussion

The use of AI and ML technologies is growing in the medical field and in particular in oncology, as testified bywith the exponential growth of publications in recent years. Our study aimed to create an explainable model to predict the response and efficacy of IO using the clinical data of patients collected at baseline IO in a real-world setting. To achieve this, we selected those features that better characterize patients diagnosed with advanced NSCLC, only using the information available in the clinical practice at the baseline of treatment to build a feasible algorithm, explainable and easily translatable for use in decision-making without increasing costs for the health system or requiring further invasive procedures.

We combined current medical literature and clinical experience with AI/ML tools to create models with a higher predictive value for the DCR (ACC=0.75) than PD-L1 alone on the same set of patients (ACC=0.56), increasing the predictive accuracy by around 25%. To reach this performance, we used five different ML models, four models previously applied in our last publication (11) with the novelty of CatBoost’s inclusion. CB achieved the best test accuracy and AUC and F1 scores for both classes for outcomes: TTF3 and OS6, while when predicting DCR as an outcome, LR and CB achieved similar results. Applying XAI methods to CB thus provides better insights into why the models performed the way they did. As reported in **Table 6**: NLR and ECOG PS appeared as the most relevant features across response and survival outcomes underling the important role of these features: while PD-L1, Line of IO, and the role of combination chemo-immunotherapy appeared to be more important in predicting DCR compared to OS6, presumably showing that high PD-L1 expression and the use of the combo therapy is more relevant to improve the response to therapy than survival. This result is crucial to underline the role of the results obtained from the KEYNOTE-189 study (6) in the subset of patients with high PD-L1 expression. Perhaps, the OS for patients treated with combo chemo-IO therapy is comparable to the ones treated with IO alone. A better DCR can be raised with the addition of chemotherapy, leading to the possible conclusion that chemotherapy boosts response while IO is the determinant in the relevant differences in survival.

Another interesting finding is that the Line of IO therapy is relevant for both DCR and OS6 outcomes, meaning that offering IO therapy in the first Line is essential for survival.

Among outcomes, ORR and OS24 are highly imbalanced, thus leading to not satisfactory results, the model is strongly biased to the mostly seen class during training. ORR as output can give useful information, so it would be interesting to tackle this problem for example by using oversampling or undersampling ML techniques, however this should be done with great precaution. Producing digital patients should be done in close cooperation among oncologists, bioengineers and data scientists. On the other hand, the PFS outcome did not have imbalanced data, but it showed lower performance compared to OS6 and TTF.

The findings of this study have to be seen in the light of some limitations: i) the database is heterogeneous, as it contains data from patients receiving IO in various lines of treatment in a real-world setting; ii) neither radiomic nor genomic features were incorporated in this analysis, thus excluding other potential biomarkers that would be relevant in the context of precision medicine.

Several studies have already reported on AI applications in NSCLC, as well as in other fields of oncology, mainly based on real-world, genomic, and radiomic data. For example, a retrospective study was conducted between 2007 and 2017 with the aim of evaluating and comparing the effects of chemotherapy, target therapy, and immunotherapy in patients with NSCLC. For each type of treatment, ORR, PFS, and OS were analyzed by ML methods, using tumor- and patient-related variables as input. Logistic Regression was the model with better performance, achieving an AUC of 0.79. The study revealed promising results for chemotherapy and target therapies, unlike for immunotherapy, possibly due to the lack of relevant predictors (26). In contrast, Lu et al. (27) integrated ML methods with whole-exome sequencing data. The authors used data from melanoma patients treated with IO to develop the model and a cohort of patients with NSCLC, also receiving IO, as a validation set. In the NSCLC cohort, the high-weight TMB group was associated with better survival and better clinical benefit at 6 months with an AUC 0.83 (27). We recently reported a combination feature algorithm using clinical, lab and microRNA signature classifier blood test to predict ICI response in NSCLC patients. Logistic Regression was used to predict responder and not responder patients with an ACC 0.756 and AUC 0.82. Long Survival patients (24-months OS) were also predicted, reporting an ACC of 0.839 (11).

As mentioned earlier, there is an increasing need to apply XAI algorithms as a *post-hoc* technique to understand each specific model and its predictions. In oncology, this need for trustfulness is even more prominent since the stakes are higher than in everyday clinical situations. One such application is the Shapley additive explanation model (SHAP) used in this study, one of the most used XAI models, which comes from game theory. **Table 7** presents a summary of the most relevant applications of XAI techniques in cancer-related research (28–35). Notably, in the present study, XAI included in the model as relevant features those clinical biomarkers that have already been shown to be important in the last 10-years of clinical research. This is an interesting demonstration in itself that the models work and it is trustworthy (36).

**Table 7:** Summary of XAI application in cancer–related research.

REFERENCES	METHOD	APPLICATION
**Yang Et al. (** 28)	Laplacian Eigenmaps	Brain tumor classification using MRS
**Zhao And Bolouri** (29)	Cluster analysis and LASSO	Lung cancer patients’ stratification
**Hao Et al.** (30)	Sparse Deep Learning	Long-term survival prediction for glioblastoma
**Suh Et al.** (31)	Shapley Value	Decision-supporting for prostate cancer
**Izadyyazdanabadi Et al.** (32)	MLCAM	Brain tumor localization
**Couture Et al.** (33)	Super-pixel Maps	Histologic tumor subtype classification
**Meldo et al.** (34)	LIME	Lung lesion segmentation
**Moncada−Torres et al.** (35)	Shap	Prediction of a breast cancer survival

## Conclusion

In conclusion, our results suggest that data integration made possible by AI techniques is a useful tool, with a high potential still, to improve prediction for NSCLC patients treated with IO. More specifically, our model shows that high NLR and ECOG PS are inversely associated with responders to IO, to patients with an OS longer than 6 month and patients with a TTF longer than 3 months. On the other hand, a high PD-L1 value together with the IO therapy in combination with chemotherapy positively correlates with DCR and TTF, while seemingly being less important for OS6 prediction. As mentioned above, integrating other biomarkers beyond PD-L1 and adapting them based on the outcome can be an attractive way to conjugate immuno-oncology and precision medicine to fine-tune these findings and deepen our understanding of response mechanisms further still.

## Data availability statement

The datasets presented in this article are not readily available because of patients' privacy protection. Requests to access the datasets should be directed to the corresponding author. The code for the CatBoost model is available at GitHub repository on the link: https://github.com/VanjaMiskovic/RW_data_IO_efficacy. The code for other ML models is available at: https://trovo.faculty.polimi.it/downloads.html.

## Ethics statement

The studies involving human participants were reviewed and approved by the Ethical Committee of “Fondazione IRCCS Istituto Nazionale Tumori. *Via* G.Venezian,1. 20133, Milan” (Apollo, INT 22_15), and all patients have signed the informed consent. It was conducted according to Good Clinical Practice guidelines and the Declaration of Helsinki principles. The patients/participants provided their written informed consent to participate in this study. Written informed consent was obtained from the individual(s) for the publication of any potentially identifiable images or data included in this article.

## Author contributions

AP: conceptualization, investigation, methodology, project administration, supervision, writing—original draft, writing—review, editing. EG: data editing, formal analysis, methodology, review, editing. VM: formal analysis, methodology, resources, software, writing—original draft, editing. MP: data curation, formal analysis, methodology, resources, software, writing—original draft, writing—review, editing. GV, BP, LM, LP, AS: data curation, methodology, investigation writing—review and editing. RM: data curation, formal analysis, methodology, writing—review, editing. AT: data curation, methodology, investigation, writing—review and editing. CP: data curation, methodology, investigation, writing—review and editing. GG: data curation, methodology, investigation, writing—review and editing. DS, CG, SM, TB: data curation, writing—review and editing. RF, MB: data curation, methodology, investigation, writing—review and editing. MO: data curation, methodology, investigation, writing—review and editing. MM: writing—review and editing. CCP, AlR, SG, MA: writing—review and editing. MCG: conceptualization, investigation, methodology, supervision, writing—review and editing. FB: supervision, writing—review, editing. MR: conceptualization, data curation, formal analysis, methodology, resources, software, supervision, validation, writing—review and editing. GR: data curation, methodology, investigation, writing—review, editing. MG: data curation, writing—review, editing. FT: conceptualization, data curation, formal analysis, methodology, resources, software, supervision, validation, visualization, writing—review, editing. ALP: conceptualization, data curation, formal analysis, methodology, resources, software, supervision, validation, visualization, writing—review, editing. ArR: data curation, formal analysis. All authors have read and agreed to the published version of the manuscript
