# Supplementary material for: Real-world data to build explainable trustworthy artificial intelligence models for prediction of immunotherapy efficacy in NSCLC patients
Source: Front Oncol. 2023 Jan 23;12:1078822. doi: 10.3389/fonc.2022.1078822 (PMC9899835; doi:10.3389/fonc.2022.1078822)
Supplement: Supplementary file 1 [file DataSheet_1.docx]

**Table S1.** Features selected for the different models

| Model | Outcome | Selected feature |
| --- | --- | --- |
| Logistic Regression  (LR) | DCR | IO/IOCT, NLR, PDL1, TNMio, ECOG PS, LDH, AMC, Age, RT, Histology |
|  | OS6 | ECOG PS, ANC, Line of IO, Liver mets, Histology, TNMio NLR AMC |
|  | TTF3 | NLR, IO/IOCT, ECOG PS, TNMio, PDL1, AMC, Histology, LDH, Liver mets |
|  | ORR | NLR, PDL, IO/IOCT, T, Surgery, N, ECOG PS, Smoke, Sex |
|  | OS24 | NLR, ECOG PS, PDL1, N, Histology, Surgery, Age, TNMd, Smoke, TNMio |
|  | PFS3 | IO/IOCT, NLR, PDL1, TNMio, LDH, BMI, Lymph nodes mets, Line of IO, Smoke, Surgery |
| Neural Network  (NN) | DCR | Line of IO, PDL, LDH, TNMio, Brain mets, RT, Surgery, ECOG PS, BMI |
|  | OS6 | ECOG PS, ANC, Liver mets, PDL1, AMC, BMI, Histology, Age, ALyC, TNMd |
|  | TTF3 | NLR, IO/IOCT, ECOG PS, BMI, PDL1, Stage, Sex, Liver mets, RT, TNMd |
|  | ORR | NLR, PDL1, Sex, IO/IOCT, T, N, Surgery, ALyC, BMI |
|  | OS24 | NLR, ECOG PS, BMI, ANC, PDL1, ALC, Sex |
|  | PFS3 | IO/IOCT, TNMio, NLR, Line of IO, PDL1, BMI, ECOG PS, N, ANC |
| Random Forest  (RF) | DCR | ALC, Line of IO, NLR, LDH, IO/IOCT, PDL1, Age |
|  | OS6 | NLR, LDH, ECOG PS, ANC |
|  | TTF3 | NLR, Age, ANC, Liver mets, Stage |
|  | ORR | BMI, NLR, PDL1, Age, IO/IOCT |
|  | OS24 | BMI, NLR, PDL1, ALC, |
|  | PFS3 | ALC, LDH, IO/IOCT, NLR, Line of IO |
| Support Vector Machine  (SVM) | DCR | ALC LDH, NLR Histology, Liver mets, Age, PDL1, AMC, ANC, Lymph nodes mets, Surgery, Line of IO, ALyC, Sex, RT |
|  | OS6 | LDH, ALC, Age, ALyC, Liver mets |
|  | TTF3 | NLR, ALC, LDH, BMI, AMC, Bone mets, Histology, Pleura mets, Smoke, Line of IO, ALyC |
|  | ORR | ALC, BMI, NLR, Age, Tumor Stage, LDH, Sex, AMC, Adrenal mets, T, ANC, Pleura mets, Bone mets, Lymph nodes mets, TNMd, TNMio, Brain mets, IO/IOCT, Line of IO |
|  | OS24 | ALC, LDH, BMI, Age, RT, NLR |
|  | PFS3 | ALC, LDH, AMC, NLR, Age, N, Liver mets, Lymph nodes mets |

**Table S2.** Performance of classification models on the test dataset; outcome - ORR

| Outcome | Model | Features | Class | n.class | Precision | Recall | F1 | Acc. | AUC |
| --- | --- | --- | --- | --- | --- | --- | --- | --- | --- |
| ORR  Class 0  360  patients  Class 1  120  patients | CB | 27 | 0 | 40 | 0.91 | 0.72 | 0.81 | 0.71 | 0.67 |
|  |  |  | 1 | 8 | 0.31 | 0.62 | 0.42 |  |  |
|  | LR | 9 | 0 | 40 | 0.84 | 0.90 | 0.87 | 0.77 | 0.75 |
|  |  |  | 1 | 8 | 0.20 | 0.13 | 0.15 |  |  |
|  | NN | 9 | 0 | 40 | 0.83 | 0.96 | 0.90 | 0.81 | 0.67 |
|  |  |  | 1 | 8 | 0.00 | 0.00 | / |  |  |
|  | RF | 5 | 0 | 40 | 0.84 | 0.90 | 0.87 | 0.77 | 0.65 |
|  |  |  | 1 | 8 | 0.20 | 0.13 | 0.15 |  |  |
|  | SVM |  | 0 | 40 | 0.84 | 0.70 | 0.77 | 0.65 | 0.39 |
|  |  |  | 1 | 8 | 0.20 | 0.38 | 0.26 |  |  |

**Table S3.** Performance of classification models on the test dataset; outcome – OS24

| Outcome | Model | Features | Class | n.class | Precision | Recall | F1 | Acc. | AUC |
| --- | --- | --- | --- | --- | --- | --- | --- | --- | --- |
| OS24  Class 0  386  patients  Class 1  94  patients | CatBoost | 27 | 0 | 41 | 0.92 | 0.80 | 0.86 | 0.77 | 0.69 |
|  |  |  | 1 | 7 | 0.33 | 0.57 | 0.42 |  |  |
|  | LR | 10 | 0 | 41 | 0.91 | 0.98 | 0.94 | 0.90 | 0.77 |
|  |  |  | 1 | 7 | 0.76 | 0.43 | 0.55 |  |  |
|  | NN | 7 | 0 | 41 | 0.87 | 0.98 | 0.92 | 0.85 | 0.87 |
|  |  |  | 1 | 7 | 0.50 | 0.14 | 0.22 |  |  |
|  | RF | 4 | 0 | 41 | 0.87 | 0.95 | 0.91 | 0.83 | 0.63 |
|  |  |  | 1 | 7 | 0.33 | 0.14 | 0.20 |  |  |
|  | SVM | 6 | 0 | 41 | 0.82 | 0.66 | 0.73 | 0.58 | 0.38 |
|  |  |  | 1 | 7 | 0.07 | 0.14 | 0.09 |  |  |

**Table S4.** Performance of classification models on the test dataset; outcome – PFS3

| Outcome | Model | Features | Class | n.class | Precision | Recall | F1 | Acc. | AUC |
| --- | --- | --- | --- | --- | --- | --- | --- | --- | --- |
| PFS3  Class 0  234  patients  Class 1  246  patients | CatBoost | 27 | 0 | 24 | 0.69 | 0.75 | 0.72 | 0.71 | 0.71 |
|  |  |  | 1 | 24 | 0.73 | 0.67 | 0.70 |  |  |
|  | LR | 10 | 0 | 24 | 0.67 | 0.83 | 0.74 | 0.71 | 0.73 |
|  |  |  | 1 | 24 | 0.78 | 0.58 | 0.67 |  |  |
|  | NN | 9 | 0 | 24 | 0.67 | 0.67 | 0.67 | 0.67 | 0.70 |
|  |  |  | 1 | 24 | 0.67 | 0.67 | 0.67 |  |  |
|  | RF | 5 | 0 | 24 | 0.59 | 0.67 | 0.63 | 0.60 | 0.66 |
|  |  |  | 1 | 24 | 0.62 | 0.54 | 0.58 |  |  |
|  | SVM | 8 | 0 | 24 | 0.54 | 0.58 | 0.56 | 0.54 | 0.54 |
|  |  |  | 1 | 24 | 0.55 | 0.50 | 0.52 |  |  |


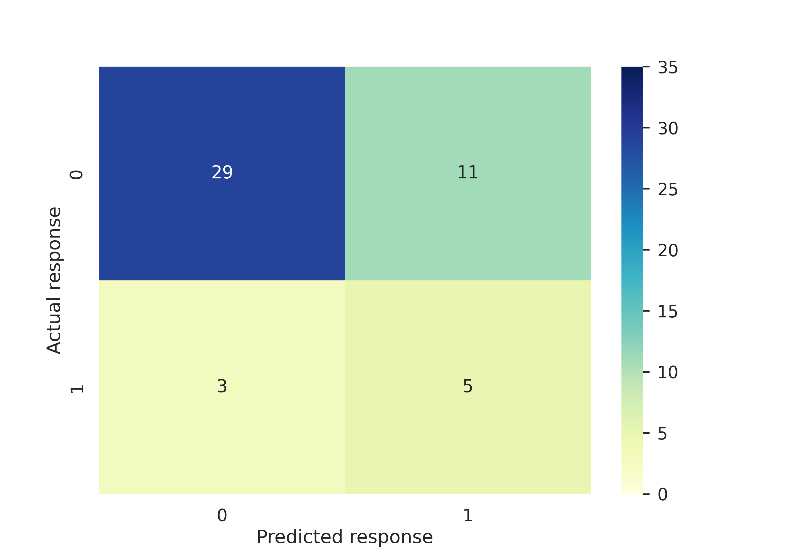

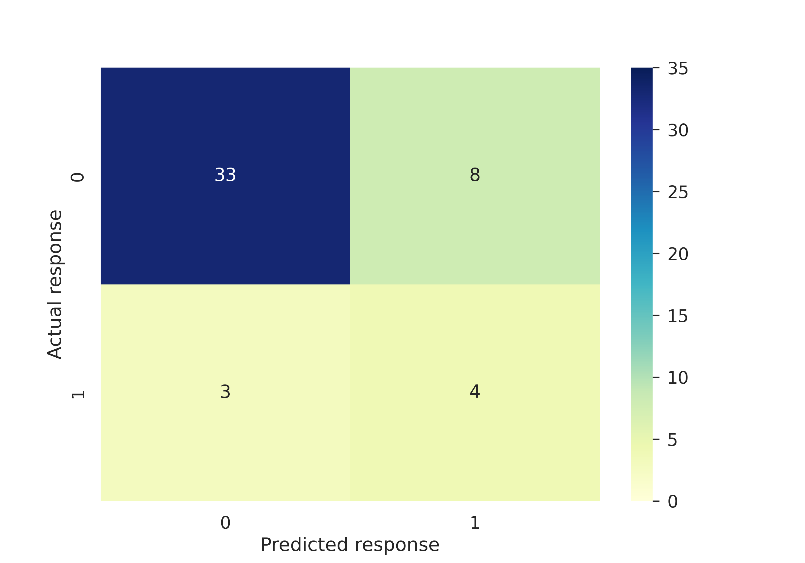


1. (b)


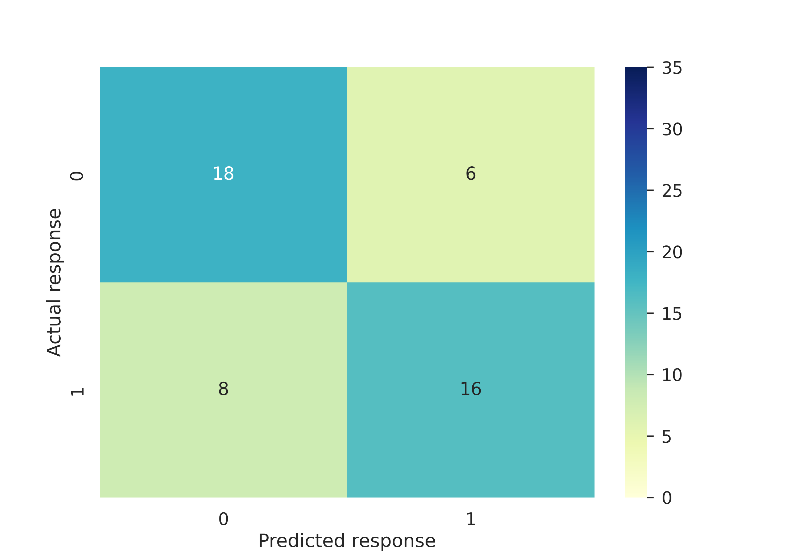


(c)

**Figure S1.** Confusion matrix for the CatBoost model for outcomes: (a) ORR, (B) OS24, and (C) PFS3


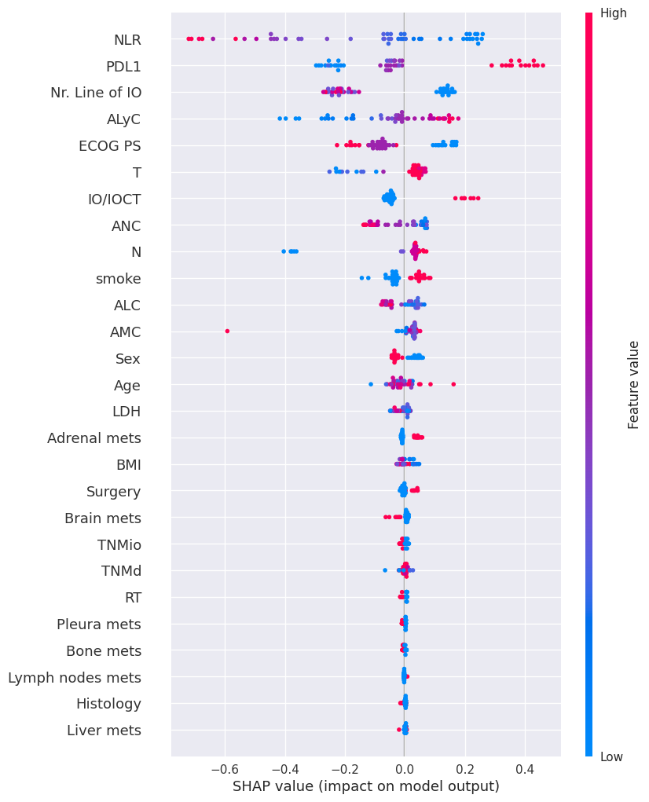

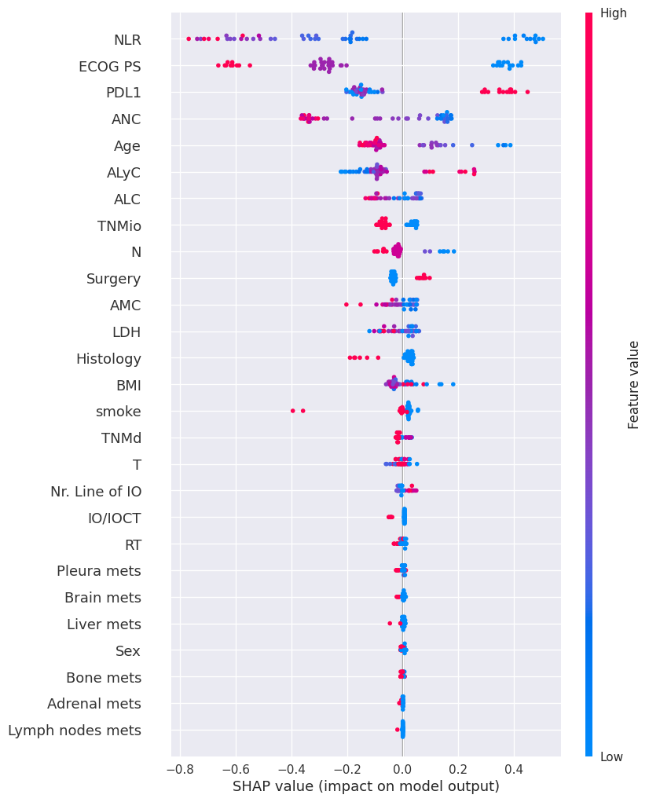


(a) (b)


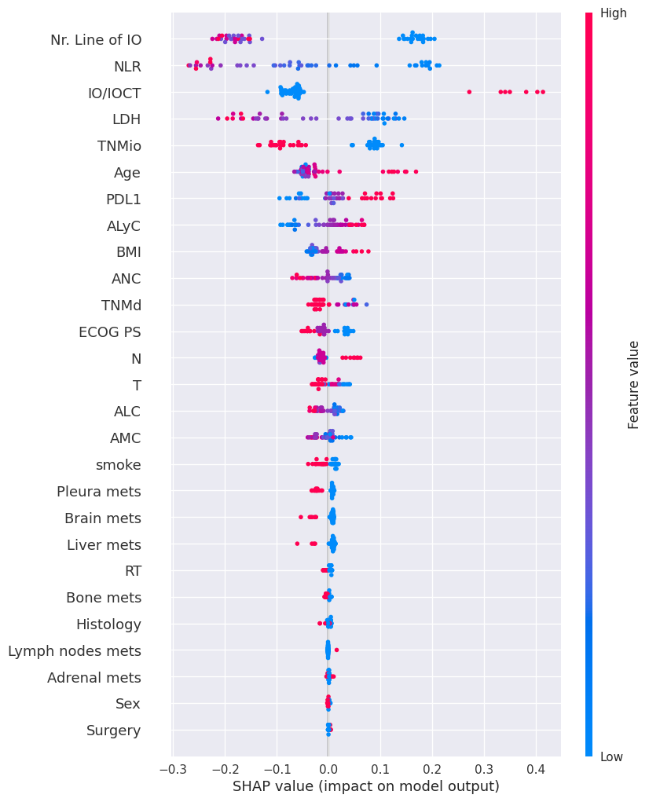


(c)

**Figure S2**. SHAP Summary plots for CB model for outcomes: (a) ORR, (b) OS24, and (c) PFS3


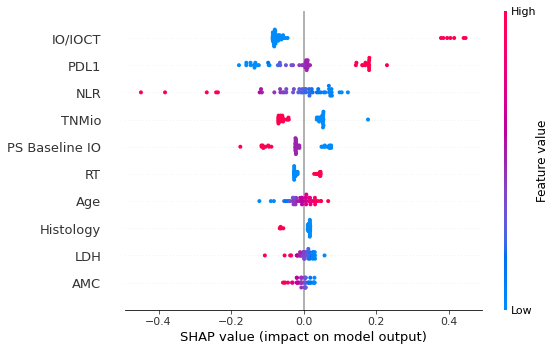

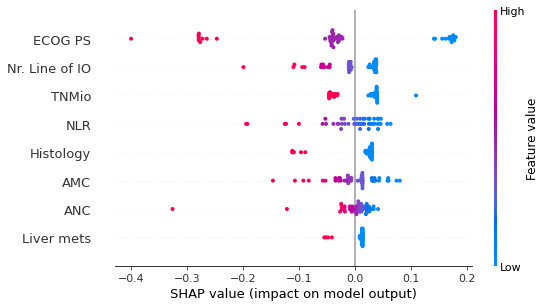


(a) (b)


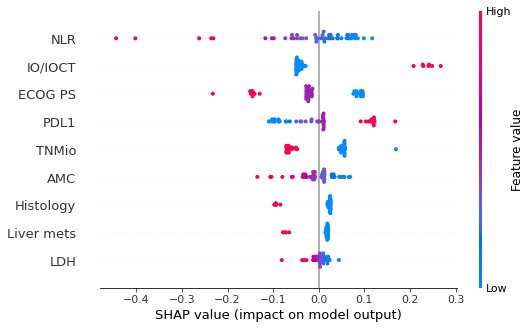

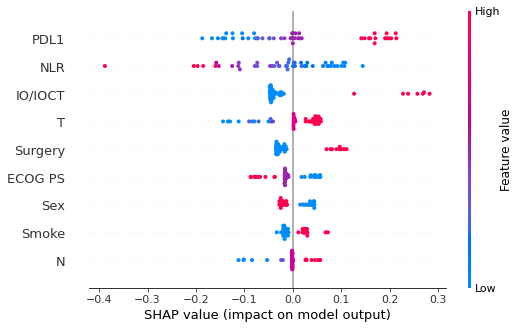


(c) (d)


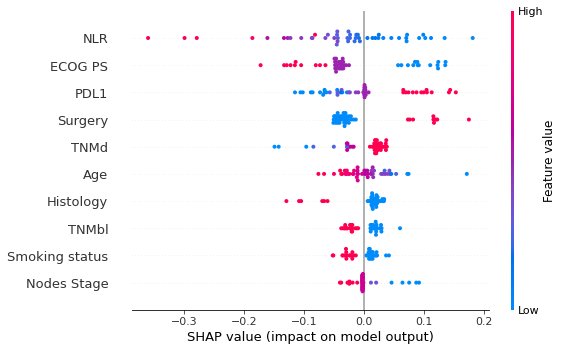

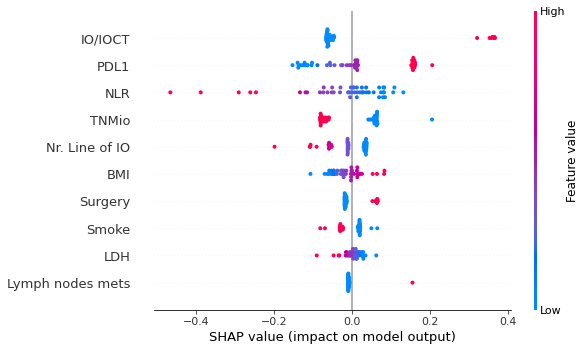


(e) (f)

**Figure S3.** SHAP Summary plots for LR model for outcomes: (a) DCR, (b) OS6, (c) TTF3, (d) ORR, (e) OS24, and (f) PFS


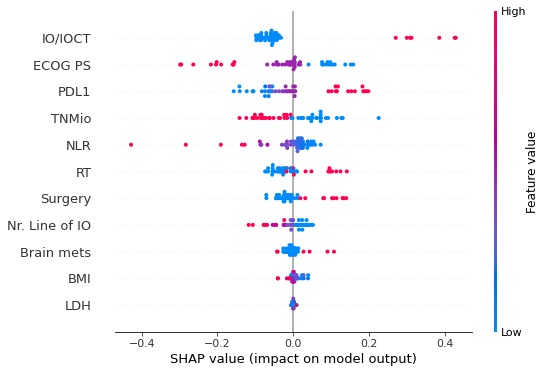

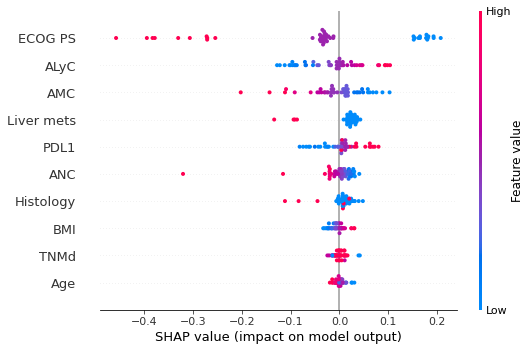


(a) (b)


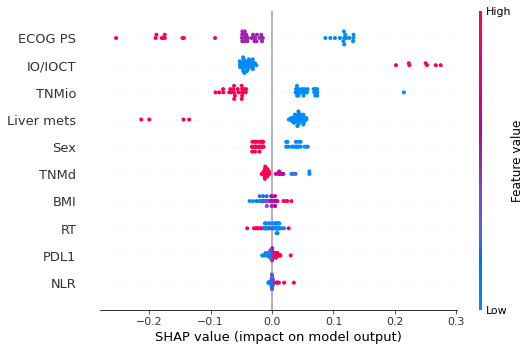

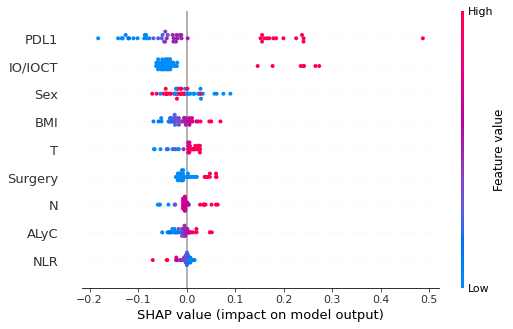


(c) (d)


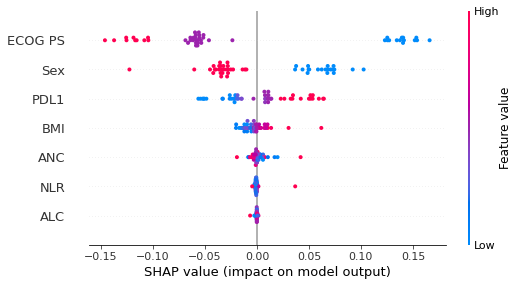

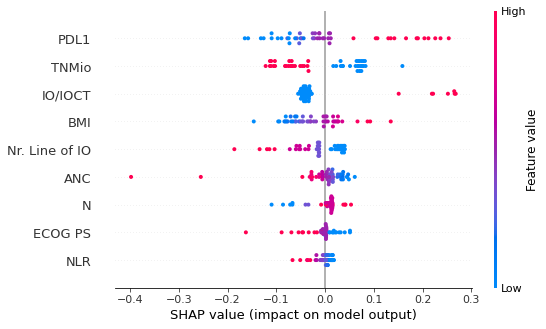


(e) (f)

**Figure S4.** SHAP Summary plots for NN model for outcomes: (a)DCR, (b) OS6, (c) TTF3, (d) ORR, (e) OS24, and (f) PFS


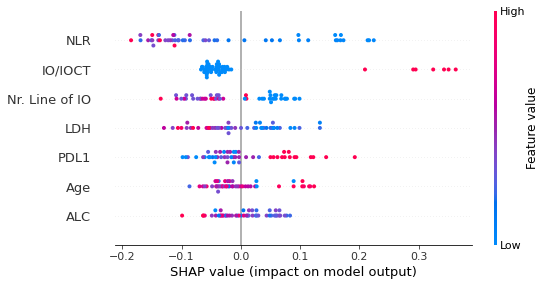

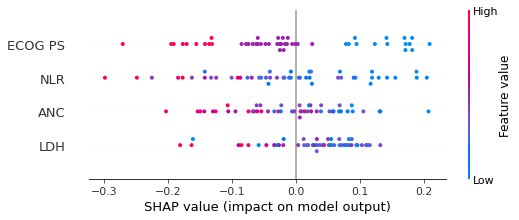


(a) (b)


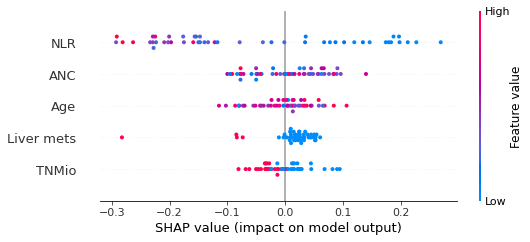

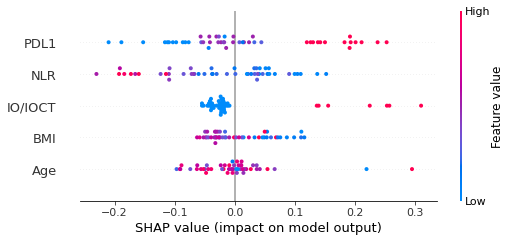


(c) (d)

**
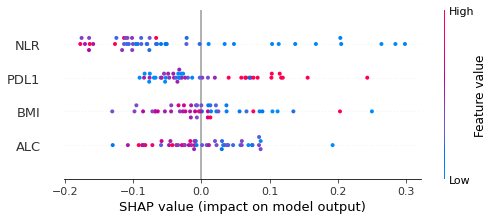

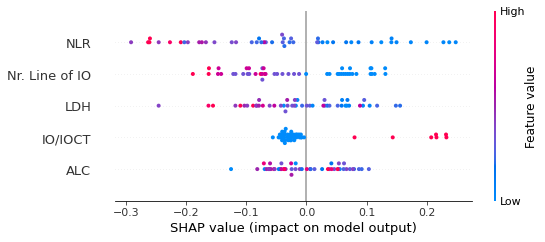
**

(e) (f)

**Figure S5.** SHAP Summary plots for RF model for outcomes: (a)DCR, (b) OS6, (c) TTF3, (d) ORR, (e) OS24, and (f) PFS


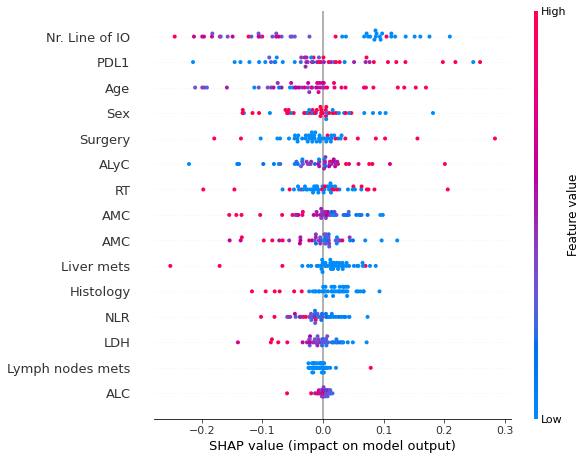

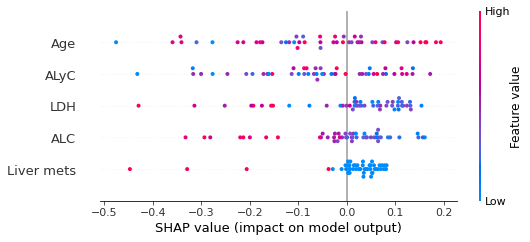


(a) (b)


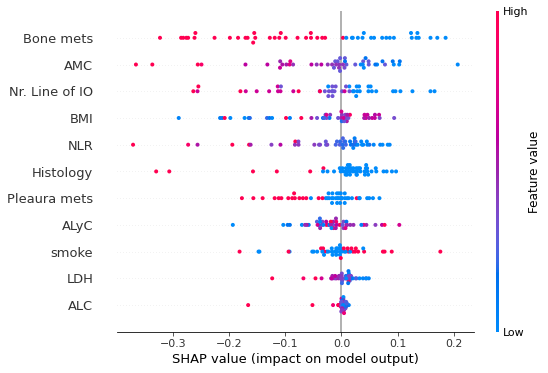

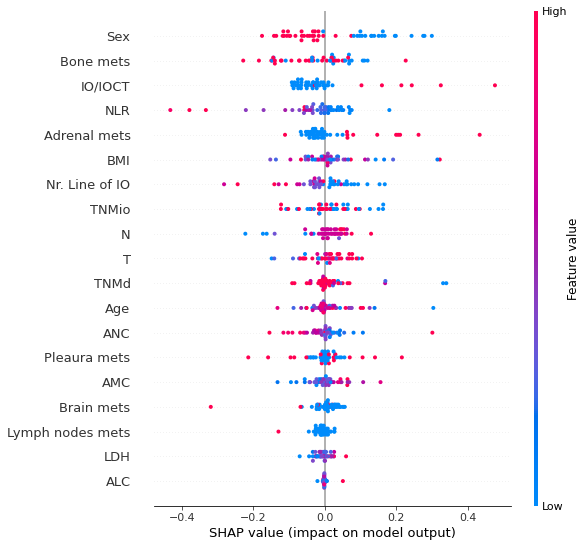


(c) (d)


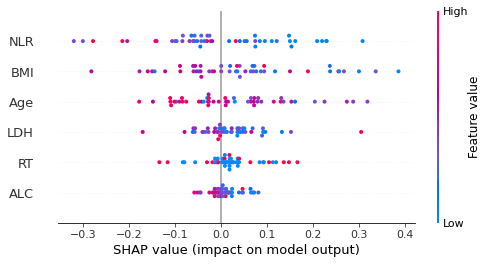

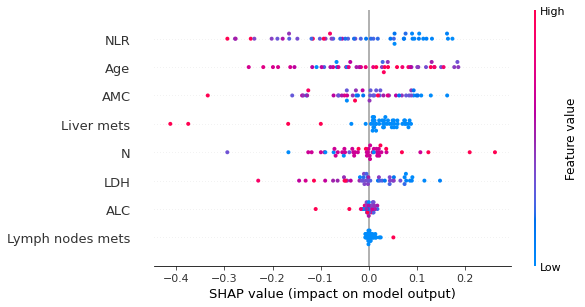


(e) (f)

**Figure S6.** SHAP Summary plots for SVM model for outcomes: (a)DCR, (b) OS6, (c) TTF3, (d) ORR, (e) OS24, and (f) PFS
